# Mesenchymal Stem Cells in Adipose Tissue and Extracellular Vesicles in Ovarian Cancer Patients: A Bridge toward Metastatic Diffusion or a New Therapeutic Opportunity?

**DOI:** 10.3390/cells10082117

**Published:** 2021-08-18

**Authors:** Gabriele Storti, Maria Giovanna Scioli, Bong-Sung Kim, Sonia Terriaca, Elena Fiorelli, Augusto Orlandi, Valerio Cervelli

**Affiliations:** 1 Plastic and Reconstructive Surgery, Department of Surgical Sciences, Tor Vergata University of Rome, 00133 Rome, Italy; valeriocervelli@virgilio.it; 2 Department of Biomedicine and Prevention, Anatomic Pathology Institute, Tor Vergata University of Rome, 00133 Rome, Italy; scioli@med.uniroma2.it (M.G.S.); terriacasonia093@gmail.com (S.T.); Elena.Fiorelli@uniroma2.it (E.F.); orlandi@uniroma2.it (A.O.); 3 Department of Plastic Surgery and Hand Surgery, University Hospital Zurich, 8091 Zurich, Switzerland; bong-sung.kim@usz.ch

**Keywords:** adipose stem cells, ovarian cancer, extracellular vesicles, drug delivery, mesenchymal stem cells, adipocytes, exosomes, micro RNA, macrophages

## Abstract

Ovarian cancer is one of the deadliest malignancies among women. Approximately 75% of the patients with ovarian cancer are diagnosed with advanced disease that already has metastasis, particularly to the omentum. The omentum constitutes the ideal soil for ovarian cancer metastasis due to a complex intraperitoneal milieu that favors and supports the whole metastatic process. Adipose-derived stem/stromal cells (ADSCs) are part of this microenvironment and foster tumor progression via sustained paracrine secretion, including extracellular vesicles (EVs). Nonetheless, the preferential relationship between ADSCs, ADSC-derived EVs, and ovarian cancer cells could be exploited to use ADSCs and EVs as a vehicle for anti-cancer therapies. This review will analyze the strict relations between tumor progression, metastatic disease, and adipose tissue with its staminal components. In addition, we will describe the crosstalk and biologic relationship between ADSCs and tumor cells, the role of EVs in intercellular communication, the establishment of drug resistance, metastatic capacity, and ovarian cancer progression. We will analyze the new therapeutic opportunities in treating ovarian cancer offered by ADSCs and EVs as a vehicle for therapeutic molecules to target precisely tumor cells and limit the systemic adverse effects. Finally, we will discuss the limitations of these therapeutic approaches.

## 1. Introduction

Ovarian cancer is one of the deadliest malignancies among women affected by gynecological tumors. Although its incidence is relatively low, accounting for only 3% of overall cancer incidence, it causes 4.4% of the cancer-related deaths, being the fifth cause of mortality among female cancer patients. The cumulative overall survival is approximately 45% at five years from diagnosis, but it drastically drops down to 25 % in most advanced stages [1]. Due to the few and nonspecific symptoms, approximately 75% of the patients with ovarian cancer are diagnosed with an advanced disease that has already presented metastatic diffusion, in particular to the omentum [2]. In these advanced patients, cytoreductive surgery is often the first option combined with chemotherapy, which is mainly platinum/taxane-based and relies on combining cisplatin or its analog carboplatin, paclitaxel, docetaxel, or doxorubicin. However, the relapse rate is high, and the therapeutic options for the relapsed disease are minimal and applicable to very selected patients [3].

The high metastatic capacity of ovarian cancer follows peculiar routes of diffusion that differ from the lymphatic and hematogenous routes of other tumors [4]. The main route of diffusion is through the peritoneal cavity to the omentum, by exfoliation and accumulation of malignant cells in the peritoneal fluid [5]. The omentum constitutes the ideal soil for ovarian cancer metastasis, according to the “seed and soil” theory described first by Paget in 1889. In particular, the current concept of the tumor microenvironment is pivotal to the understanding of the metastatic process [6]. The tumor microenvironment is constituted by a specific biological niche where multiple cellular subtypes and cancer cells interact together, and with the surrounding extracellular matrix (ECM), through multiple signaling pathways, ultimately favoring cancer progression and metastasis [7]. Nowadays, one of the most plausible mechanisms of ovarian cancer cells diffusion is transperitoneal, via exfoliation of cancer cells, as single cells or multicellular clusters, which are anoikis-resistant. These exfoliated cells disseminate in the peritoneal cavity and reach, via the ascitic effusion, the omental fat pad where they adhere and invade the submesothelial tissues, creating new metastatic foci [8]. A complex intraperitoneal milieu favors and supports the whole metastatic process. It includes the original tumor bulk, the exfoliated cells, clusters in the ascitic fluid, and a plethora of resident cells in the adipose and peritoneal tissues, including fibroblasts, adipocytes, macrophages, plasmacytoid dendritic cells, mesenchymal stem/stromal cells (MSCs), infiltrating lymphocytes, and others. Furthermore, it has been hypothesized that one of the reasons behind the preference of metastatic ovarian cells for the omentum is the abundant adipose tissue [9]. 

Omental adipose tissue contains many adipose-derived stem/stromal cells (ADSCs), which can foster proliferation migration and chemoresistance of ovarian cancer cells [10]. ADSCs are a population of MSCs present in the adipose tissue throughout the whole body. MSCs have been first discovered by Friedenstein in the bone marrow, and they have been progressively characterized phenotypically [11]. Three main features have been identified to classify MSC populations: (1) plastic adherence in standard culture conditions; (2) positivity for the expression of CD105, CD90, and CD73, and negativity for CD34, CD45, CD14 or CD11b, CD79α, or CD19 and HLA-DR surface molecules; and (3) potential to undergo trilineage differentiation (adipogenic, chondrogenic, and osteogenic) [12]. In 2001, P. Zuk and colleagues have described, for the first time, an MSC population in the adipose tissue, which was identified as ADSCs [13]. ADSCs are embedded in the ECM and located in a perivascular niche shared with different cell types, including macrophages, lymphocytes, vascular endothelial cells, smooth muscle cells, fibroblasts, and pericytes. All these cellular populations taken together are named stromal vascular fraction (SVF), attainable via collagenase digestion of the ECM in the adipose tissue. ADSCs account for about 20% of the SVF and have a close relationship with the other SVF cells regulated by a high paracrine activity, based on several mediators [14]. Local and general conditions, such as obesity, inflammation, sepsis, ischemia-reperfusion injury, tissue repair, and cancer, influence the secretory activity of ADSCs in the microenvironment [15,16,17,18]. 

The crosstalk between ADSCs and their microenvironment is regulated by a sustained paracrine secretion of several mediators, including growth factors, lipids, siRNAs, lncRNAs, miRNAs, and DNA. Many of these factors are encapsulated into the so-called extracellular vesicles (EVs) [19]. The EVs are spherical bodies surrounded by bi-layered phospholipid membranes released by several cell types, including tumor cells, to deliver paracrine signals to other target cells. EVs are classified according to their dimensions into three main categories: exosomes that range between size of 40 and 150 nm and have their origin in the endosomal system; microvesicles that derive from outward blebbing of the plasma membrane and have a size between 50–3000 nm; and apoptotic bodies which are only released during the last step of apoptosis and have a size between 800 and 5000 nm [20]. In the primary tumor microenvironment and in the niche of omental metastasis, EVs secreted in the extracellular space are the main actors of the communications between tumor cells, ADSCs, and other cell types, such as macrophages and immune cells, ultimately favoring cancer invasion, progression, and drug resistance [21]. Moreover, tumor cells can recruit MSCs from the surrounding adipose tissue and even from the bone marrow [22] to receive metabolic support and survival advantage, conditioning ADSCs and the whole tumor microenvironment through a sustained secretion of EVs [23]. Although this close relationship between omental adipose tissue, ADSCs, and tumor cells seems to favor cancer progression, the tendency of tumor homing of ADSCs and their high secretory capacity could be exploited for delivering therapies directly inside the tumor microenvironment [17]. Furthermore, the EVs secreted by the ADSCs could be a good vehicle for drugs or bioactive molecules such as miRNAs, siRNAs, lncRNAs, cytokines. Thus, the use of EVs could be a potential off-the-shelf, cell-free therapeutic option for ovarian cancer.

This review will analyze the strict relations between tumor progression, metastatic disease, and adipose tissue with its staminal components. In particular, we will discuss the role of the omental and visceral fat that is a fundamental factor in understanding the biology of ovarian cancer. We will describe the crosstalk and biologic relationship between ADSCs and tumor cells in the tumor microenvironment, the role of EVs in intercellular communication, and the establishment of drug resistance, metastatic capacity, and ultimately ovarian cancer progression. We will analyze the new therapeutic opportunities in treating ovarian cancer derived from the possible clinical application of ADSCs and EVs as a vehicle for therapeutic molecules to target precisely tumor cells and limit the systemic adverse effects. Moreover, we will discuss the preclinical studies investigating these therapeutic approaches and the possible limitations derived from the use of ADSCs and ADSC-derived EVs for ovarian cancer treatment.

## 2. Ovarian Cancer and Its Metastatic Pathway 

As mentioned above, ovarian cancer is often discovered at an advanced stage, already presenting intraperitoneal diffusion and multiple metastases. Most of the ovarian cancers originate from the epithelial cells and can be roughly classified into five main subtypes: low-grade serous carcinoma, high-grade serous carcinoma, clear cell carcinoma, endometrioid carcinoma, and mucinous carcinoma [24]. High-grade serous carcinoma is the most common subtype, accounting for 75% of epithelial ovarian cancers. It is often asymptomatic, and it rapidly progresses to advanced-stage disease. 

**Figure 1:**
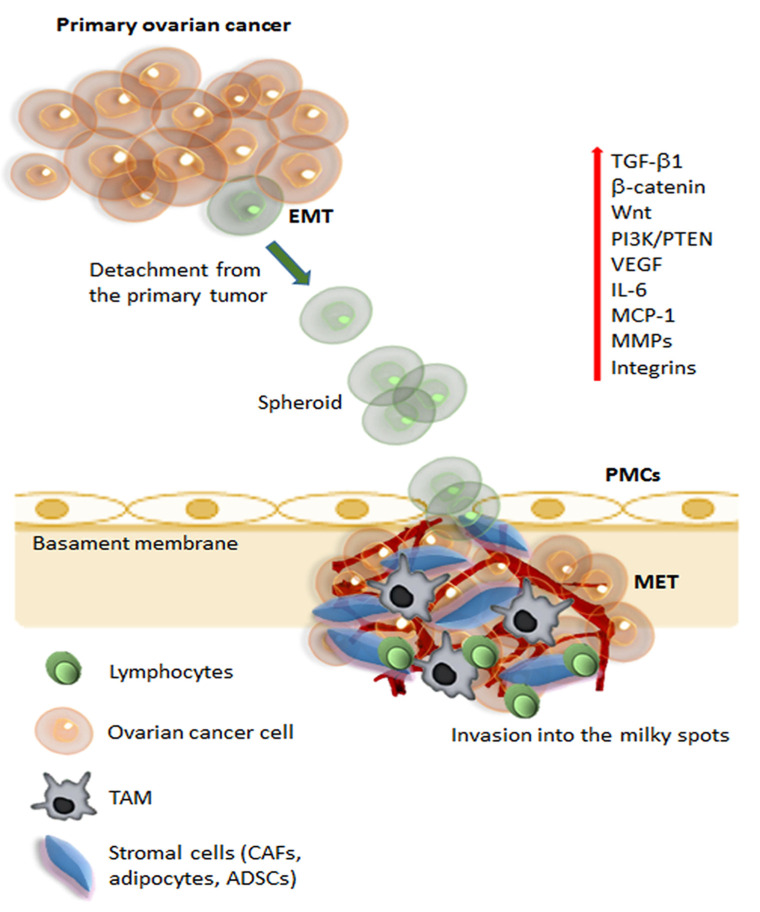
Schematic representation of metastatic pathways of ovarian cancer. The initial steps of metastasis required several mechanisms, including suppression of adaptive immune response, ECM reorganization, and the promotion of epithelial-mesenchymal transition (EMT). During initial tumorigenesis, ovarian carcinoma cells undergo EMT, which involves a change in integrin expression and up-regulation of different signaling pathways (Wnt, β-catenin, PI3K, IL-6), angiogenic (VEGF), and proteolytic molecules (MMPs). Cancer cells can detach from the tumor and, carried by the peritoneal fluid, cancer cell spheroids overcome anoikis and attach preferentially on the abdominal peritoneum. Cell aggregates find preferential engraftment into the omental “milky” spots, composed of lymphocytes, macrophages (TAMs), and stromal cells (CAFs, adipocytes, and ADSCs). At the peritoneal interface, cancer cells invade peritoneal mesothelial cells (PMCs) facilitated by integrins and TGFβ1, released by tumor cells and CAFs. Ovarian cancer cells undergo MET (reverse EMT) to acquire an epithelial phenotype, enabling the cells to establish and grow, developing secondary tumors and metastasis. Abbreviations: ECM, extracellular matrix; EMT, epithelial-mesenchymal transition; VEGF, vascular endothelial growth factor; MMPs, matrix metalloproteinases; TAMs, tumor-associated macrophages; CAFs, cancer-associated fibroblasts; ADSCs, adipose-derived stem/stromal cells; PMCs, peritoneal mesothelial cells; TGFβ, transforming growth factor-β; MET, mesenchymal-epithelial transition.

Ovarian cancer presents a specific dissemination route, which does not follow the classical lymphatic and hematogenous routes, but often proceeds through intraperitoneal dissemination. Unlike other cancers such as breast, prostate, colorectal, lung, and liver cancers, ovarian cancers do not follow the classical metastatic cascade. The metastatic cascade is a complex process that involves several steps, including local invasion, penetration in the local vessels and survival of the tumor cells in circulation, hematogenous spreading and colonization at distant metastatic sites after extravasation, cell survival, and proliferation [25]. Many of these steps are rate-limiting and require that cancer cells overcome several environmental challenges to survive and proliferate, thus prolonging the time to metastasis. On the contrary, ovarian tumors do not have anatomical barriers that separate the primary ovarian tumor from the intraperitoneal microenvironment. Furthermore, ovarian cancer cells have a specific tropism for the adipose-rich microenvironment in the omentum [26]. Transcoelomic diffusion follows precise steps, summarized in four main passages (Figure 1) [21].

First of all, single ovarian cancer cells or cell clusters exfoliate into the peritoneal cavity. This first passage is facilitated through the development of an aggressive phenotype in the tumor cells that undergo EMT and lose cell-to-cell contact. The exfoliated cells have acquired the ability to survive in the peritoneal cavity and escape anoikis and immune surveillance. If the floating cells survive, they can reach distant organs in the peritoneal cavity, showing a specific preference for the adipose-rich microenvironment of the omental fat pad. In particular, the so-called “milky spots” have been identified as one of the preferred metastatic locations for ovarian cancer cells [9]. The milky spots are aggregates of immune cells, including macrophages, B and T lymphocytes, MSCs, and adipocytes organized into a capillary network with a glomerular-like structure. Macrophages in the milky spots seem to have a chemotactic role in the recruitment of floating ovarian cancer cells and are possible contributors to the rapid colonization of milky spots [27]. 

In the phase of omental colonization, the complex interplay between the adipocytes, MSCs, and omental macrophages in the milky spots plays a crucial role in establishing metastatic localization. Macrophages are conventionally divided into two main subpopulations called M1 and M2, which diverge in their differentiation status and role in the immune system. An unbalance between the M1 and M2 population, in favor of M2, seems to be one of the factors responsible for ovarian cancer metastatic progression [28]. The macrophages that infiltrate into the tumor milieu are defined as tumor-associated macrophages (TAMs) [29]. TAMs present an M2-polarized phenotype and have a high paracrine secretion that has a fundamental role in establishing the immunosuppressive tumor microenvironment, which increases tumor growth, angiogenesis, invasion, and further metastatic diffusion [30]. The ADSC-mediated secretion of IL-6 has a central role in promoting the M2-polarization of TAMs. It has been demonstrated in a mouse model that IL-6 secreted by the ADSCs from omental adipose tissue recruited monocytes, driving their differentiation toward an M2-phenotype [31]. The shift toward an M2 phenotype in the macrophage population also promotes the macrophage-mediated synthesis of VEGF, and TGF-β1, which have an immunosuppressant effect, enhance tumor vascularization, and promote EMT and tumor progression [32]. ADSCs in the omentum can also recruit monocytes/macrophages from distant sites via the secretion of chemotactic molecules, such as MIP- 2, and MCP-1 [31]. EVs have also been identified as a possible culprit for M2-polarization and the formation of TAMs. In particular, adipocytes in the tumor microenvironment secrete MVs containing miR-21, which induces the differentiation of tissue macrophages toward M2-TAMs [33]. Parallelly, TAMs also have a paracrine secretion of miR-21, which promotes tumor growth via silencing the tumor suppressor gene PTEN [34]. 

All these mechanisms collaborate with the metastatic tumor cells to establish a new metastatic focus. Ovarian cancer cells undergo MET (reverse EMT) to acquire an epithelial phenotype enabling the cells to establish and grow, developing secondary tumors and metastasis. Finally, the tumor cell proliferation and invasion determine the growth of the tumor burden, which is sustained by adipocytes and ADSCs through several molecular pathways, as described more thoroughly below. 

## 3. The Relationship between Ovarian Cancer, Adipose Tissue, and Its Staminal Component

For a long time, adipose tissue was considered an inert tissue with the sole functions of energy storage and thermal isolation. Nonetheless, over the years, an increasing burden of evidence demonstrated how the adipose tissue has an important endocrine role that regulates many physiological and pathological processes, including weight regulation, inflammation, and cancer. Therefore, the consideration of adipose tissue shifted toward indicating adipose tissue as a proper endocrine organ with a complex organization. The main cellular actors in the adipose tissue are adipocytes and ADSCs, which have continuous crosstalk with ovarian tumor cells, particularly those contained in the omental and visceral adipose tissue. Obesity has been listed as a risk factor for many types of cancer, including gastrointestinal, mammary, renal, and reproductive cancers in both sexes [35]. Obesity is a risk factor for the development of ovarian cancer, and it has been related to a higher risk of cancer progression and reduced overall survival [36]. Understanding the relationship between adipose tissue cells, the tumor cells, and the tumor microenvironment is pivotal to focus on possible therapeutic targets and tumor escape mechanisms from the available therapies.

### 3.1. Adipocytes in the Ovarian Cancer Microenvironment

Adipocytes are the cell type that constitute most of the volume in the adipose tissue. Adipocytes seem to have a strict metabolic, endocrine, and paracrine relationship with cancer cells. Adipose tissue can be roughly divided into white adipose tissue (WAT), brown adipose tissue (BAT), and a third type that has been recently identified and termed beige adipose tissue, which has features between WAT and BAT. WAT and BAT have different functions in the body and different metabolic profiles. WAT is located in the whole body and mainly in two main deposit areas: surrounding internal organs, which is defined as visceral WAT; and between the muscles and the superficial fascia, which is termed as superficial WAT [22]. The visceral WAT has the role of protecting the internal organs from traumas, while subcutaneous WAT has the function of energy storage. Conversely, BAT is a highly oxidative tissue that produces heat through the lipid oxidization via the mitochondrial uncoupling protein-1 (UCP1) action and is highly represented in the early phases of human life [37]. UCP-1 is located in the inner mitochondrial membrane and promotes thermogenesis by uncoupling mitochondrial ATP synthesis and cellular respiration. WAT has a high secretory activity, which is enhanced in conditions of low-grade inflammation such as cancer and obesity [38]. 

#### 3.1.1. Adipocyte-Mediated Paracrine Secretion in the Tumor Microenvironment

It has been demonstrated that omental adipocytes secrete several molecules, including adipokines, such as leptin and adiponectin, and several pro-inflammatory cytokines. The levels of leptin are directly proportional to the total amount of fat present in the body. High leptin levels have been associated with reduced progression-free survival in Middle Eastern women with epithelial ovarian cancer [39]. Several mechanisms have been investigated to explicate the role of leptin in ovarian cancer progression. Leptin acts on a transmembrane receptor called Ob-R/LEPR, which could be present also in ovarian cancer cells. It has been demonstrated, on ovarian cancer cell lines, that the activation of the Ob-R determined an inhibition of apoptosis and an increased cell proliferation via the upregulation of the expression of cyclin D1 and myeloid cell leukemia-1 (Mcl-1), which are apoptosis regulators [40]. Furthermore, Ob-R can upregulate downstream the JAK/STAT3, PI3/AKT, and RhoA/ROCK signaling pathways, ultimately leading to the promotion of the epithelial-mesenchymal transition (EMT) and contributing to the maintenance of a staminal phenotype in tumor cells [41]. Moreover, the blockade of the leptin receptor has been demonstrated to inhibit tumor progression through the diffusion of ovarian cancer cells in the peritoneal cavity in an animal model [42]. Therefore, adipocyte-secreted leptin could be a target for potential therapies of ovarian cancer.

The low-grade inflammation present in cancer patients stimulates adipocytes to secrete inflammatory cytokines. Nieman and colleagues demonstrated that the co-culture of omental adipocytes with ovarian cancer cells SKOV3 stimulates homing, migration, and invasion of ovarian cancer cells, in vitro and on animal models [43]. In addition, the authors demonstrated an increased secretion of IL-6, IL-8, TIMP1, and MCP1, which was responsible for the pro-oncogenic effects of adipocytes. Cytokine secretion determines a promotion of angiogenesis, tumor invasion, and the chemotaxis toward the omentum of tumor cells and other cell types, such as macrophages or MSCs [38]. 

#### 3.1.2. The Metabolic Interplay between Omental Adipocytes and Tumor Cells

Ovarian cancer is also able to influence the omental and visceral adipose tissue toward a metabolic shift. One of the phenomena that have been evidenced in patients with cancer is the “browning” of the WAT, which is the acquisition of BAT’s features [28]. The elevation of IL-6 has been linked to the “browning” phenomenon and could be one of the reasons for cancer cachexia development. The elevation of IL-6 levels increases the transformation of WAT into BAT, thus increasing energy consumption and ultimately leading to cachexia. In the early phases of cancer cachexia, the inhibition of IL-6 can revert the browning of the WAT in a mouse model [44]. In ovarian cancer patients, the perirenal adipose tissue seems to be more prone to the browning phenomenon, and an increased thickness of this fat pad has been proposed as a marker of a reduced progression-free survival [45].

It has been demonstrated that omental adipocytes contribute directly to the tumor cells’ metabolism by transferring free fatty acids. The co-culture of adipocytes and ovarian cancer cells determines increased lipolysis in omental adipocytes, which is coupled to an upregulation of β-oxidation in cancer cells [43]. These metabolic changes suggest that the adipocytes act as an actual energy source for the omental metastatic cancer cells. One of the proposed mechanisms that sustain this metabolic exchange is the increased expression of fatty acid-binding protein 4 (FABP4) in metastatic ovarian cancer cells at the interface with omental adipocytes [43]. FABP4 is a low-molecular-weight protein that has the role of transporting long-chain fatty acids and other hydrophobic ligands. The expression of FABP4 is higher in metastatic ovarian cancer than in primary tumors, and it has been proposed as a tumor marker for metastatic disease. FABP4 synthesis in the adipocytes seems to be a critical step for fatty acid transfer from adipocytes to cancer cells, and has a role in angiogenesis and tumor proliferation [46]. FABP4 has been proposed as a therapeutic target, and FABP4 elevation in primary ovarian cancer tissue was significantly associated with the risk of residual disease after primary debulking surgery in patients with high-grade serous carcinoma [47].

Another proposed mechanism underlying the metabolic relation between cancer cells and adipocytes is the activation, via autophosphorylation, of the salt-inducible kinase 2 (SIK2), a member of the AMP-activated protein kinase (AMPK) family. At the cancer-adipose tissue interface, adipocytes of the omental tissue induce the calcium-mediated activation of SIK2 in ovarian cancer cells. SIK2 activation and enhanced expression determine an increase in fatty acid oxidation by activating the transcription of carnitine palmitoyltransferase 1 (CPT1) and increasing the AMPK-induced phosphorylation of acetyl-CoA carboxylase (ACC). Furthermore, SIK2 promotes cancer cell proliferation and metastatic progression via the activation of the PI3K/AKT pathway [48]. Moreover, part of the adipocytes directly interacting with ovarian tumor cells in the tumor microenvironment undergoes a process of dedifferentiation into preadipocytes, acquiring a more immature phenotype.

#### 3.1.3. Cancer-Associated Adipocytes (CAAS)

The relationship between omental adipocytes and cancer cells is so intense that the adipocytes undergoing the secretory and metabolic shift mentioned above have been renamed cancer-associated adipocytes (CAAs). CAAs have a specific phenotype that includes lower lipid content, an overexpression of proteases and inflammatory cytokines, and a reduced expression of late adipose markers [49]. As described above, CAAs have high secretory activity. For example, CAA secretion of IL-8 stimulates the tumor cell homing and invasion into the omental fat pad [43]. Moreover, CAA secretion also includes EVs. In particular, microvesicles containing miR-21 are secreted by CAA and uptaken by ovarian cancer cells. The intra-cytoplasmatic release of miR-21 in cancer cells downregulates the mRNA for apoptotic peptidase activating factor 1 (APAF-1). APAF-1 plays a pivotal role in the apoptosis process, being a part of the apoptosome and triggering apoptosis initiation via the autocatalytic activation of procaspase-9 mediated by the cytochrome-c. Furthermore, APAF-1 has a rate-limiting function in this apoptotic pathway, essential in taxane-induced cell death. Therefore, miR-21-mediated resistance to apoptosis seems to be responsible for paclitaxel resistance [50]. 

### 3.2. Adscs in the Ovarian Cancer Microenvironment

**Figure 2:**
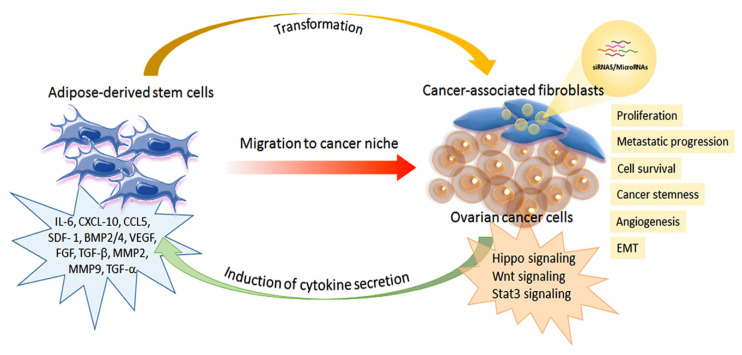
Schematic representation of the interplay between adipose-derived stem cells and ovarian cancer cells.

As described above, ADSCs are a fundamental component in the tumor stroma, both in the primary tumor and in the adipose-rich omental metastatic niche. Over the years, ADSCs have gained increasing attention for their role in supporting tumor growth and progression [51]. Many authors have demonstrated that ADSCs, and particularly those from omental adipose tissue, can increase ovarian cancer cell proliferation and promote a shift toward a more aggressive, invasive, and metastatic phenotype [10,31,52,53] (Figure 2).

#### 3.2.1. From Adscs to Cancer-Associated Fibroblasts (CAFs)

The role of mesenchymal stem cells is fundamental for the omental metastatic microenvironment of ovarian cancer. In particular, omental ADSCs can induce tumor angiogenesis via VEGF and SDF1-α secretion, thus increasing ovarian cancer cell survival. Moreover, ADSCs stimulate ovarian cancer cell proliferation and metastasis by producing MMP2 and MMP-9 proteins. In addition, ADSCs can secrete different inflammatory and proangiogenic factors, including IL-1 receptor antagonist, IL-6, IL-10, CCL5, VEGF, and MMP-2. These factors are involved in ovarian cancer metastatic invasion. The presence of ADSCs in the cancer microenvironment and their transformation in cancer-associated fibroblasts (CAFs) is essential to promote ovarian cancer growth, survival, epithelial-mesenchymal transition (EMT), and the acquisition of a cancer stem cell like-phenotype. CAFs can induce upregulation in the TGF-β/BMP family, thus favoring the invasiveness of ovarian cancer cells. In addition, CAFs can produce various tumor-promoting factors, such as IL-6, SDF-1α, and VEGF-A, in the metastatic tumor microenvironment. TGF-β secretion can promote ovarian cancer metastasis, which stimulates CAFs to secrete cytokines and chemokines, including IL-6, CXCL10, and CCL5, that favor cancer cell metabolism and energy production. Altogether, ADSCs and then CAFs in the ovarian cancer microenvironment and their interplay regulate cancer cell adhesion, survival, proliferation, and metastatic progression. Furthermore, ovarian cancer cells can recruit ADSCs and MSCs to support their growth and induce a phenotypic shift toward a myofibroblastic phenotype, thus assuming the characteristics of cancer-associated fibroblasts (CAFs) [54,55]. Therefore, the intense relationship between ovarian cancer cells and ADSCs is bidirectional and involves several biological mechanisms that have been investigated in vitro and animal models.

Several molecular mechanisms have been described to explain the pro-proliferative and pro-invasion effects of ADSCs on ovarian cancer cells. Chu et al. found an upregulation of the mRNA coding for PAX 8, which was associated with an activation of the hippo signaling pathway in ovarian cancer cells [56]. Increased levels of PAX8 raised the levels of TAZ by stabilizing it and preventing its degradation; TAZ is a transcriptional coactivator upregulating cell proliferation. The PAX8-mediated pro-proliferative effect was also confirmed in vivo in a mouse model. Another proposed mechanism is the upregulation of the Thymosin Beta 4X-Linked (TMSB4X) expression in ovarian cancer cells. ADSC-mediate enhanced expression of TMSB4X promoted ovarian cancer growth and metastasis both in vitro and in an animal model [57]. A proteomic profile of ovarian cancer cells demonstrated an increase in TMSB4X expression in ovarian cancer cells after exposure to an ADSC-conditioned medium. TMSB4X is an actin-binding protein with anti-apoptotic effects that regulates hypoxic reaction and controls cancer cell migration. TMSB4X inhibition canceled the pro-metastatic effects of ADSCs on ovarian cancer cells.

#### 3.2.2. ADSC Secretory Activity and Its Effects on Ovarian Cancer Cells 

MSCs in general, including ADSCs, have demonstrated a high paracrine secretory activity in response to inflammation and wound healing processes. Besides, the tumor has been described as a “non-healing wound” [58], and the tumor microenvironment could be considered an inflammatory setting. The low-grade inflammation that is present in ovarian cancer stimulates ADSC secretion of multiple inflammatory cytokines. IL-6, CXCL-10, CCL5, SDF- 1, BMP2/4, VEGF, FGF TGF-β, MMP2, and MMP9 are among the pro-tumoral cytokines secreted by ADSCs [48]. Co-culture of human omental ADSCs and SKOV3 ovarian cancer cells increased cancer cell proliferation and migration via an increased ADSC secretion of MMP-9 and MMP-2. The favoring effect in metastasis was also confirmed in a mouse xenograft model [52]. Thus, MMPs can regulate pathways such as Hedgehog, Wnt, PI3K/Akt, and NF-κB in cancer cells. Moreover, Zhang et al. reported that ADSC-conditioned medium could also increase MMPs’ synthesis as a result of an alteration in the proteomic profile of ovarian cancer cells [59]. The authors inferred an EV-mediated mechanism and found an upregulation of the mRNA, PHB, and SRSF-1 in the ovarian cancer cells treated with the ADSC-conditioned medium. PHB mediates MMPs expression, thus regulating apoptosis, cancer cell proliferation, and migration. SRSF-1 is a splicing factor that promotes the transformation, proliferation, and survival of cancer cells via enhanced survivin expression. Furthermore, SRSF1 could determine β-catenin accumulation and the activation of the Wnt signaling pathway.

IL-6 is one of the most important mediators of ADSC effects on ovarian cancer. Omental adipose tissue in cancer patients presents an increased IL-6 production. IL-6 can recruit ADSCs in the tumor site and induce the EMT in the cancerous cells [19]. Moreover, the ADSCs recruited and located in the tumor microenvironment have the capacity of increasing IL-6 paracrine secretion by themselves. ADSC-produced IL-6 has been demonstrated to be responsible for increased autophagy and migratory capacity in ovarian cancer cells [60,61]. A study by Chu and colleagues demonstrated that the exposition of ovarian cancer cells to omental-ADSC-conditioned medium increased the level of autophagy [60]. Autophagy is a survival behavior typical of rapidly growing cancers, and consists of a cell self-degradation process that allows reusing metabolites, such as fatty acids, nucleotides, and amino acids. Therefore, autophagy allows tumor survival even in hypoxia or high metabolic needs, such as in rapid growth of the tumor burden. The authors of this preclinical study demonstrated that high levels of IL-6 contained in the ADSC-conditioned medium activated the STAT3 pathway, which is linked with the promotion of tumor growth and invasion and is responsible for the increase in autophagy in ovarian cancer cells.

IL-6 also enhances the migration capacity of SKOV3 ovarian cancer cells. Kim and coworkers demonstrated that the IL-6 contained in the conditioned medium from cultures of visceral and subcutaneous ADSCs activated the JAK2/STAT3 signaling pathway in SKOV3 cells, thus enhancing their migratory and proliferative capacity [61]. Thus, blocking the activation of JAK2 and STAT3 via inhibitors, siRNAs, or neutralizing IL-6 could suppress ovarian cancer cell migratory capacity, making them possible targets for future therapeutic approaches. In this study, no difference in tumor promotion between visceral and subcutaneous tissue ADSCs was noticed. Nonetheless, other authors have described significant differences in ovarian cancer/ADSC interactions when analyzing subcutaneous or visceral ADSCs. Nowicka et al. compared the tumor-promoting capacity of omental ADSCs both from metastatic and non-metastatic patients, subcutaneous ADSCs, and bone marrow MSCs (BM-MSCs) [10]. They demonstrated that omental ADSCs favor ovarian cancer cell proliferation and migration more than subcutaneous ADSCs. In particular, ADSCs from metastatic patients have a particular phenotype that had stronger tumor-promoting effects when compared to non-metastatic omental ADSCs, subcutaneous ADSCs, and BM-MSCs. Moreover, omental ADSCs promoted ovarian cancer chemoresistance and radiation resistance.

#### 3.2.3. ADSC-Mediated Genetic and Metabolic Effects on Ovarian Cancer Cells 

Omental ADSCs from metastatic patients had a different gene expression profile with changes in expression of genes coding for membrane proteins and proteins secreted in the extracellular space, thus further demonstrating the high paracrine activity. The main pathways involved included cell-to-cell signaling, cell migration and proliferation, and cytokine-mediated signaling pathways (TNF and IL-8 pathways). In addition, in a mouse xenograft model, omental ADSCs could engraft into cancer-affected ovaries but not into normal ones, demonstrating a tumor-specific engraftment process. The effect of omental ADSCs on cell proliferation differed among ovarian cancer cell populations. While ADSCs had a pro-proliferative effect on OVCA 429, OVCA 433, and SKOV3 cell lines, they determined a negative effect on A2780 cell proliferation. Similarly, another group described an anti-proliferative effect of omental ADSCs co-cultured with A2780 and SKOV3 ovarian cancer cells [62]. The ADSCs-conditioned medium determined a downregulation of the anti-apoptotic gene BCL-2 and upregulation of pro-apoptotic genes such as BAX, CASP3, and CASP9 in ovarian cancer cells. Thus, the authors proposed an exosome-mediated effect. In particular, ADSC-derived exosomes seemed to act through a miRNA-mediated mechanism, and the authors proposed a hyperexpression of anti-cancer miRNAs, such as overexpression of hsa-miR-124-3p, which dysregulates different CDKs arresting the cell in the S-phase. However, the specific factors regulating a pro-proliferative or anti-proliferative phenotype of ADSCs have not been fully understood yet. 

Besides the visceral or subcutaneous origin, another factor that could affect ADSCs behavior is the metabolic status of the patient, precisely a condition of obesity. In an animal model, Zhang and colleagues demonstrated that obesity increased the tumor-promoting capacity of ADSCs from the subcutaneous tissue, which reaches that of visceral ADSCs [31]. Visceral ADSCs and subcutaneous ADSCs from obese mice had similar effects in tumor promotion and increased the growth of intraperitoneal ID8 tumors compared to controls when injected intraperitoneally. Furthermore, increased secretion of MCP-1, MIP-2, and IL-6 also mediated a chemotactic role in increasing the infiltration of macrophages and inflammatory cells into the tumor.

Omental ADSCs are also responsible for metabolic changes in the tumor microenvironment, which seem to be mediated by nitric oxide (NO) [63]. NO has a role in tumor progression and metastasis, and omental ADSCs can regulate NO balance in the tumor microenvironment, thus supporting cancer growth, metastasis, and chemoresistance. In addition, ADSCs can secrete arginine and rescue tumor cells from arginine-deprived conditions, thus allowing cancer cells to synthesize NO. Furthermore, ADSC production of NO increases the glycolytic pathway in tumors and inhibits the cellular respiratory chain. Exosomes derived from MSCs can transfer a new capability to ovarian cancer cells based on metabolizing 5’-AMP into adenosine through the acquisition of the ecto-5’-nucleotidase activity [64]. This newly acquired metabolic capacity allows cancer cells to suppress and modulate pro-inflammatory activities and have an immunomodulating function.

#### 3.2.4. The Effects of Tumor Cells on ADSC Recruitment and ADSC Phenotypic Changes

As much as omental ADSCs have a paracrine secretory activity supporting ovarian tumors, cancer cells can recruit ADSCs and MSCs and send paracrine signals to direct their behavior and change their phenotypic characteristics. ADSCs are attracted to the tumor site and have a specific tumor homing due to several chemotactic factors in the tumor microenvironment. In particular, in the omentum of ovarian cancer patients, high levels of CXCL-10 and CCR5 have been described [53]. Furthermore, recruited ADSCs secreted IL-10, which has a strong chemotactic activity for Treg lymphocytes and could play a role in immunosuppression in the tumor microenvironment. Another molecule implicated in the chemotactic recruitment of multipotent MSCs is the LL-37 peptide, the C-terminal peptide of human cationic antimicrobial protein 18. LL-37 stimulates MSC recruitment from distant sites to the omentum. Furthermore, it induces MSC secretion of several pro-inflammatory and pro-angiogenic factors, including IL-1 receptor antagonist, IL-6, IL-10, CCL5, VEGF, and MMP-2 [65]. Ovarian cancer cell secretory activity can transform the ADSCs in cancer-associated ADSCs (ca-ADSCs) with a paracrine mechanism [66]. This specific population, isolated from ovarian tumors’ stroma, has an increased staminal phenotype and promoted tumor growth more efficiently than non-neoplastic ADSCs do. Similar phenotypic changes could be induced in standard ADSCs by exposure to ovarian cancer cell-conditioned medium. Furthermore, after a few passages, the tumor conditioned medium is no longer necessary, thus indicating a possible autocrine loop. Ca-ADSCs also have a specific genetic and secretory profile with an increased expression and secretion of BMP2/4, which can partly justify their increased efficiency in tumor growth and may also promote the formation of microcalcification.

Numerous authors have hypothesized that omental ADSCs, recruited in the tumor site, could function as precursor cells of several cell types present in the tumor stroma. In particular, ovarian cancer cells can recruit ADSCs and BM-MSCs and induce them to acquire a myofibroblastic phenotype and function as cancer-associated fibroblasts (CAF). CAFs have a fundamental role in supporting the tumoral growth and secrete in a paracrine way several molecules such as chemokines (CXCL12), IL-6, TGF-β, and miRNAs, for example miR-21, which downregulates APAF-1 and reduces apoptosis, as mentioned above [67]. Mechanisms underlying MSC and ADSC transformation in CAFs are under thorough investigation. Tang and coworkers highlighted that the co-culture of ADSCs and epithelial ovarian cancer cells could induce the phenotype shift in ADSCs with the expression of myofibroblastic phenotype markers, such as α-SMA and fibroblast-activated protein expression [55]. The authors proposed a TGF-β1-mediated mechanism that promoted the phenotypic shift and increased the capacity of ovarian cancer cells to proliferate and invade. The same effects were also confirmed in vivo. Interestingly, the authors demonstrated that these effects were present in ADSCs from patients affected by ovarian cancer with or without omental metastasis, but not in patients with benign ovarian diseases. Parallelly, tumor exosomes have been considered responsible for this phenotypic shift in ADSCs with different mechanisms depending on the investigated cell line, in vitro [23]. An increased expression of SDF-1, TGF-β, and TGF-β receptors I and II was described in all the exosome-treated cells, but with different pathways. Downstream the TGF-β receptors, SKOV3-cell exosomes activated a SMAD-dependent pathway, while OVCAR-3-cell exosomes acted through the hyperphosphorylation of AKT. Finally, ADSC-derived CAFs secrete TGF-β, which establishes an autocrine loop and act on tumor cells, thus promoting EMT [67].

Soluble factors in the ascitic fluid could also be responsible for the shift of ADSCs toward a CAF phenotype. Lysophosphatidic acid (LPA) is one of the most studied. LPA secreted by cancer cells in the ascitic fluid can induce CAF transformation in ADSCs via hyperexpression of SDF-1 and autocrine activation of the TGF-β1-SMAD pathway [54]. Furthermore, LPA induces ADSCs to secrete VEGF, which has a robust pro-angiogenetic role. Rho-kinase, ERK, PLC, and PI3K are other pathways implicated in the LPA-mediated differentiation of ADSCs into CAFs. Kidd and colleagues made a quantitative analysis of the mesenchymal staminal components in the ovarian tumor microenvironment. They tried to distinguish between adipose-derived MSCs and bone marrow-derived MSCs in tumor stroma [22]. Interestingly, they described that different subpopulations of CAFs have different origins. MSCs from bone marrow sources constituted most of the FSP-positive and FAP-positive CAFs. In contrast, most vascular and fibrovascular stroma (pericytes, a-SMA+ myofibroblasts, and endothelial cells) originated from adipose tissue close to the tumor site. 

Finally, the relationship between MSCs and cancer cells could determine increased drug resistance. ADSCs can attenuate the cisplatin-induced apoptosis in ovarian cancer cells through a reduced intracellular cisplatin accumulation and a reduction in the caspase 3 [68]. As mentioned above, the transfer from cancer-associated adipocytes and ADSC-derived CAFs of exosomal miR-21 could induce paclitaxel resistance through an APAF1- mediated mechanism [50]. Other miRNAs that have been found in EVs and linked to platinum resistance are miR-21-3p, miR-21-5p, and miR-891-5p [69]. Furthermore, drugs such as cisplatin can promote the secretion of EVs from cancer stem cell-rich spheroids. These EVs, released in response to cisplatin, can stimulate the migration of MSCs and increase the secretion of IL-6, IL-8, and VEGFA, thus stimulating a more aggressive and tumor-promoting phenotype in MSCs [70]. Considering ADSC preferential tumor homing, their high secretory capacity, and all the ADSC-dependent mechanisms in tumor development and progression, ADSCs and their EVs could be considered a promising starting point for developing new biological therapies.

## 4. ADSCs and ADSC-Derived EVs as Therapeutic Strategies for Ovarian Cancer

Even though ADSCs, particularly those from the omentum, seem to have a relevant role in tumor promotion and progression, many of their features could be exploited for therapeutic purposes. As described above, ADSCs tend to home into the tumor microenvironment, responding to paracrine chemotactic signals from tumor and other stromal cells. Furthermore, they have a high paracrine secretory activity and are easy to manipulate in culture. These features could be exploited to transform the ADSCs into vehicles for drugs or bioactive molecules with a tumor-suppressing capacity (Table 1).

### 4.1. EV-Mediated Antitumor Effects and Possible Therapeutic Strategies

As discussed above, the relationship between ADSCs and ovarian cancer seems to have mainly tumor-promoting effects. Nonetheless, some authors have also demonstrated a possible tumor-inhibiting role of ADSCs and their EVs [10,62,72,73]. Omental ADSCs, from both metastatic and non-metastatic patients, could reduce the proliferation of A2780 ovarian cancer cell lines in vitro [10]. Similar results could be obtained using the ADSCs’ culture medium in SKOV3 and A2780 cell cultures. Therefore, a paracrine mechanism has been hypothesized. 

ADSC-released exosomes have been proposed as the vehicle of tumor-suppressing miRNAs. This miRNA-mediated mechanism increased pro-apoptotic molecules (BAX, CASP9, and CASP3), whereas decreased the levels of the anti-apoptotic protein BCL2 [62]. ADSC-secreted microvesicles from immortalized ADSCs demonstrated the capacity to induce pro-apoptotic and necrotic effects in two ovarian cancer cell lines, in vitro [73]. The administration of ADSC-derived microvesicles determined a dramatic reduction in the tumor cell secretion of pro-tumorigenic cytokines, such as IL-6, IL-8, GRO-alpha, and VEGF. On the other hand, microvesicles increased the levels of tumor-suppressive cytokines such as IL-1ra, IL-2, IL-2Ra, IL-12-p40, IL12-p70, IL-15, and IFN-γ. Similar results have been obtained with microvesicles derived from different mesenchymal sources, such as BM-MSCs. BM-MSC microvesicles inhibited SKOV3 cell growth and proliferation, both in vitro and animal models [81].

Khalil and coworkers studied the ovarian cancer-inhibiting capacity of MSCs from a different source, including adipose tissue [72]. They demonstrated that the co-culture of several ovarian cancer cell lines, both with MSCs and their conditioned media, determined a reduction in the level of CA-125 and LDH, along with increased apoptosis. Moreover, the MSC-conditioned medium significantly decreased the invasive capacity and aggressiveness of cancer cell lines. These effects were mediated by a decrease in MMP-2, MMP-9, and CA-125 mRNA expression. The cytokine profile was also modified with an increase in IL-4 and IL-10 and an IL-6 reduction. However, the specific factors influencing the development of a pro-tumorigenic or anti-tumorigenic MSC phenotype have not been understood yet. Cho and colleagues demonstrated that the conditioned medium from hyperthermia-conditioned ADSCs has a higher tumor-suppressive capacity [71]. Thus, the authors hypothesized that hyperthermia (43 °C for 45 min) could enhance the expression of tumor-inhibiting mRNAs by ADSCs.

miRNAs contained in MSC-secreted EVs have been demonstrated to reduce resistance to chemotherapy. For example, EVs containing miR-181 can inhibit ovarian cancer cells’ chemoresistance [82]. miR-181 reduces chemoresistance via the downregulation of MEST, thus inactivating the Wnt/β-catenin signaling pathway. Furthermore, MSC-derived exosomes containing miR-146 increased the sensitivity of ovarian cancer cells to taxane and docetaxel [83]. miR-146 targeted laminin γ2 (LAMC2), reducing its expression and activation of the PI3K/Akt pathway, thus increasing chemosensitivity. Another miRNA that could be a candidate for tumor treatment is miR-424. MSC-derived EVs containing miR-424 reduced tumor cells’ proliferation and angiogenic capacity, decreasing VEGF through downregulation of the MYB pathway [84]. Thus, all the miRNAs mentioned above could be future therapeutic strategies if overexpressed by ADSCs or delivered by EVs to the tumor site. 

Kobayashi and colleagues have explored the possibility of creating engineered exosomes to deliver tumor-inhibiting miRNAs [85]. They selected miR-199a-3p as a possible candidate for suppressing tumor growth. Engineered exosomes loaded with miR-199a-3p were efficiently internalized by several ovarian cancer cell lines and reduced cell proliferation in vitro and omental dissemination in the animal model. c-Met is a direct target of miR-199a-3p, and its reduced expression downregulated the ERK pathway and MMPs secretion.

### 4.2. Drug Loaded ADSCs

Another possible therapeutic strategy that exploits ADSCs could be the preloading of ADSCs with chemotherapeutic agents and their use as a drug vehicle. Drug-loaded ADSCs have proven to be useful in other oncological settings, such as breast cancer [86]. One of the advantages of this approach is the preferential homing of ADSCs and ADSC-derived EVs, which determines a direct delivery of the drug into the tumor site, thus reducing the minimal efficacious dose, systemic toxicity, and protecting the drug from early degradation.

Melzer et al. used an immortalized population of MSCs incubated with different chemotherapeutic agents, including taxol and epirubicin [74]. MSCs efficiently internalized drug molecules and released them into exosomes in the extracellular spaces. Drug-loaded exosomes have a higher cytotoxic effect at 1000-fold lower concentrations than free taxol in vitro. Moreover, they demonstrated a high tumor-suppressing capacity, both on primary tumor and distant metastases, when administered intravenously in the animal model. 

Borghese and coworkers demonstrated that ADSCs primed with paclitaxel could release the drug and inhibit Paclitaxel-resistant ovarian cancer cells’ growth [75]. The paclitaxel-ADSC-conditioned medium demonstrated a similar tumor-inhibiting capacity. In both cases, i.e., for cells and conditioned medium, the cytotoxicity was higher than with free paclitaxel and could overcome the resistance to paclitaxel, both with 2D and 3D culture systems (heterospheroids). Furthermore, paclitaxel-ADSCs aggregated together with ovarian cancer cells to form heterospheroids and inhibit their migratory and proliferative capacity. This tendency of ADSCs to aggregate with ovarian cancer cells in 3D structures could be exploited for intraperitoneal chemotherapy to interfere with floating cancer cells.

### 4.3. Genetically Modified ADSC

Genetical modification of ADSCs is another promising strategy to treat ovarian cancer, which has been evaluated in numerous studies. For example, ADSCs and MSCs could be modified to express anticancer molecules such as interferon (IFN)-β or tumor necrosis factor-related apoptosis-inducing ligand (TRAIL) or express prodrug-converting enzymes in the tumor site.

#### 4.3.1. ADSC-Based Enzyme/Prodrug Systems

Prodrugs are non-active anti-cancer agents activated in the tumor via an enzymatic conversion to the active form. This approach could allow selective activation of the drug in the tumor site, thus increasing local concentrations and reducing systemic toxicity. In addition, ADSCs could be genetically modified to express the converting enzyme and carry it to the tumor microenvironment.

Malekshah and colleagues tested an enzyme prodrug system in ovarian cancer intraperitoneal metastasis, both in vitro and in the animal model [77]. They selected irinotecan as a possible chemotherapeutic agent to be delivered intraperitoneally. The conversion of irinotecan to its highly cytotoxic form SN-38 is achieved by the human carboxylesterase-2 (shCE2) enzyme. Nonetheless, systemic administration of irinotecan at doses that allow killing drug-resistant tumor cells could be highly toxic and can cause life-threatening side effects. Irinotecan co-administered with shCE2-expressing ADSCs inhibited the proliferation of ALDH+, drug-resistant, ovarian cancer cells at far lower concentrations than other chemotherapeutic drugs tested in vitro. Furthermore, shCE2-ADSCs, after intraperitoneal injection, localized in the tumor stroma and also in the necrotic zones, in a mouse model. The possibility to target ALDH^+^ cells, which often correspond to cancer stem cells, and to arrive in areas such as the necrotic zones, where drug diffusion is poor, and there often is a high concentration of drug-resistant cells, enhances the therapeutic potential of shCE2-ADSCs. Furthermore, the coadministration of irinotecan with shCE2-ADSCs allowed eradicating ovarian cancer in 80% of the treated mice, which remained cancer-free for the rest of the study, using lower doses of irinotecan and reducing systemic toxicity.

Another enzyme/prodrug tested to treat ovarian cancer cells is the combination of yeast cytosine deaminase: uracil phosphoribosyltransferase (yCD:UPRT) and 5 fluorocytosine (5-FC). yCD:UPRT enzyme converts 5-FC to 5-fluorouracil (5-FU), which is the active form [87]. After the administration of yCD:UPRT-expressing MSCs, 5-FC could efficiently kill SKOV3 ovarian cancer cells both in vitro and in an animal model, without evident toxicity. Furthermore, the 5-FU obtained by 5-FC conversion efficiently killed the MSC clones, thus limiting the oncological risk correlated to long-term engraftment of the MSCs. This enzyme /prodrug administration system is under evaluation in patients with high-grade gliomas (NCT02015819 and NCT01172964). 

Another study by Toro et al. showed that ADSCs can be engineered either to express yCD:UPRT or Herpes simplex virus thymidine kinase (HSVtk) [76]. The HSVtk can convert the prodrug ganciclovir to its toxic metabolites. The authors evidenced an excellent efficacy of the HSVtk-expressing ASDCs/ganciclovir combination on SKOV3 cells but not on A2780 cells. On the contrary, the yCD:UPRT-expressing ADSCs/5-FC combination was efficacious both in 2D and 3D culture systems and both on SKOV3 and A2780 cells. Furthermore, they have observed that 3D culture systems were good predictors of in vivo response. yCD:UPRT-expressing ADSCs/5-FC combination achieved long-term tumor-free survival in 33% of the animals. Interestingly, the administration of yCD:UPRT-expressing ADSCs alone had a relevant tumor-supporting effect, highlighting one of the possible risks of these cell-based therapies. A clinical trial investigating the combination of HSVtk-expressing MSCs and ganciclovir has been carried out, but no results are available at the moment (NCT00005025).

#### 4.3.2. ADSC-Mediated Expression of Tumor-Suppressing Molecules or Oncolytic Viruses

The use of viral plasmids has also been attempted to obtain ADSC and MSC overexpression of tumor-suppressing molecules such as TRAIL and IFN-β. TRAIL is a cytokine which has demonstrated promising anti-cancer features. Nonetheless, its use has been limited often by low stability and inefficient delivery in the tumor site [88]. Kuroki and colleagues used a TRAIL-based molecule called TR3 and transduced the TR3 expressing gene to ADSCs via an adenoviral vector [79]. Transduced ADSCs efficiently expressed TR3 and secreted it in the extracellular space. Therefore, transduced ADSCs and their conditioned medium could inhibit ovarian cancer cell growth in vitro. Yin and coworkers attempted another strategy to enhance the ADSC expression of TRAIL [78]. They used magnetic nanoparticles to deliver a heat-inducible gene vector that encoded for TRAIL into ADSCs. As a result, mild magnetic hyperthermia induced TRAIL expression in ADSCs, which efficiently killed ovarian tumor cells in vitro. Furthermore, intraperitoneal injection of TRAIL-expressing ADSCs in a mouse model of metastatic ovarian cancer resulted in preferential homing of ADSCs in the tumor and efficient TRAIL delivery with a significant decrease in tumor volume. 

IFN-β overexpressing MSCs have been proposed as another possible option for ovarian tumor treatment [89]. MSCs transduced with an IFN-β-carrying adenoviral vector were recruited in the tumor microenvironment after intraperitoneal injection in a mouse model and efficiently expressed IFN-β. Thus, complete tumor eradication was observed in 70% of the treated mice, while an increased survival was noted in all the treated mice. Currently, a clinical trial is investigating the tolerability of IFN-β-expressing MSCs as an intraperitoneal injection in ovarian cancer patients (NCT02530047).

Finally, the combination of ADSCs and an oncolytic virus, such as the Measles virus, has been used to treat ovarian cancer in vitro and on an animal model [80]. ADSCs efficiently delivered the Measles virus to the tumor stroma, obtaining a cancer-cytotoxic effect and significantly prolonged survival in the treated animals. The tolerability, feasibility, and efficacy of this approach is currently under evaluation in a phase I/II clinical trial in ovarian cancer patients, whose results are expected in 2022 (NCT02068794).

### 4.4. Advantages and Limitations of Therapeutic Approaches Using ADSC and ADSC-EV 

The use of ADSCs and specifically omental ADSCs could present several advantages. ADSCs have demonstrated a higher proliferative capacity than MSCs from other sources and maintain good genetical stability after numerous passages in culture. Furthermore, the number of MSCs in one gram of fat could be 2,500-fold higher than in bone marrow [12]. Moreover, omental fat could be easily harvested during primary oncologic surgery, and the obtained ADSCs could be successfully expanded in vitro. Other advantages are the preferential tumor homing and the high secretory activity, making them good candidates for drug or gene delivery systems.

Even though all these positive aspects favor ADSCs use as a cell-based cancer therapy, some limitations could hinder their translation into clinical practice. First of all, the possible oncogenic risk. The permanent engraftment of ADSCs could ultimately favor tumor development. Furthermore, a theoretical risk of genetic senescence of cultured cells with the development of pro-oncogenic mutations exists, even though it has not been described yet for ADSCs. Another possible limitation is the expansion and manipulation of ADSCs and transduction with viral vectors, which can pose regulatory limitations and impair production upscaling. Lastly, the biodistribution of the ADSCs is not yet clear [90]. After intravenous injection, ADSCs seems to remain trapped mainly in the filter organs, the lungs, and the liver. Therefore, intraperitoneal injection is probably the best administration route in ovarian cancer but implies a more invasive procedure with a potential higher risk of complications. Another possible critical aspect of the ADSC-based therapies for ovarian cancer is the interactions with TAMs. TAMs have been demonstrated to internalize drug-loaded microparticles efficiently, thus allowing them to reach TAMs and enhancing their therapeutic efficacy [91]. However, it has been demonstrated that even heat-inactivated ADSCs maintain immunomodulatory properties, orienting the monocyte differentiation toward an M2 phenotype [92].

The use of EVs could overcome many of these limitations. For example, EVs have a similar capacity as their cellular counterpart to deliver their cargo directly to the tumor site and protect it from degradation. Furthermore, their reduced dimensions could allow them to pass the lungs and be delivered to the target sites even after intravenous injection. Moreover, they do not have an oncologic risk or a risk of permanent engraftment or transformation. Finally, their low immunogenic potential allows a possible allogeneic use, making it easier to develop off-the-shelf products [93]. However, the main limitation of the use of ADSC-derived EVs consists in the production upscaling. Standardizing EV contents and obtaining a product without impurities is extremely difficult with significantly high costs, impairing translation into clinical practice [20].

## 5. Conclusions

Ovarian cancer is one of the most challenging neoplastic diseases that unfortunately has a high risk of metastatic diffusion and a low chance of obtaining therapeutic success. Most of the therapeutic chances rely on surgery, while medical therapies have poor outcomes. 

As we have discussed, adipose tissue and, in particular, ADSCs have an ambivalent relationship with ovarian cancer. The close vicinity of omental fat and omental ADSCs to the tumor could boost disease progression and metastasis. ADSC-secreted factors and EVs have a central role in pivotal steps of tumor growth, such as cell proliferation, resistance to apoptosis, EMT, migration, invasion, and drug resistance. However, the preferential homing of ADSCs and ADSC-secreted EVs could offer the possibility to target cancerous foci, limiting systemic toxicity and delivering therapies to the hardest-to-reach cancer sites and drug-resistant cells. ADSCs and ADSC-EVs could act as trojan horses to reach the sanctum sanctorum of the tumor, where standard therapies would diffuse poorly or reach too low concentrations. Nonetheless, even though strategies employing ADSCs and ADSC-derived EVs could be promising, their translation in everyday clinical practice has many obstacles to face. Therefore, more preclinical studies are needed to define the safety and efficacy of these therapies as much as their evaluation in patients in large-scale clinical trials.

## Figures and Tables

**Table 1:** Possible ADSC-based therapeutic strategies for ovarian cancer Abbreviations: ADSC: adipose-derived stem cells; CM: conditioned medium; Exo: exosomes; MV: microvesicles; PTX: Paclitaxel; IRI: irinotecan.

Authors and Year of Publication	Possible Therapeutic Agent	Possible Mechanism of Action	Experimental Model	Results	Reference
Direct Cellular Inhibition					
Cho, J.A., 2009	CM from Hyperthermia-conditioned ADSCs	Reduced expression of mRNAs for MDR1 and Bfl-1 (Bcl-2 family). Increased expression of mRNA coding for TNF-R	In vitro on SKOV3 cell cultures	CM from Hyperthermia-treated ADSCs had enhanced suppressive effect on tumor progression and malignancy compared to normal ADSC controls	[71]
Reza, A.M.M.T., 2016	CM from ADSCs	Exo-mediated reduction in expression of anti-apoptotic BCL-2 and increased levels of pro-apoptotic BAX, CASP9, and CASP3, exo-miRNAs-mediated CDK dysregulation	In vitro on SKOV3 and A2780 cell cultures	reduced proliferation, cell viability, wound-repair capacity, and colony formation ability of A2780 and SKOV-3 ovarian cancer cells	[62]
Khalil, C., 2019	ADSCs from subcutaneous lipoaspirate and their CM	Decrease in MMP-2, MMP-9, and CA-125 mRNA expression. The cytokine profile was modified with an increase in IL-4 and IL-10 and an IL-6 reduction.	In vitro, co-culture of OVCAR3, CAOV3, IGROV3 and SKOV3 with ADSCs and ADSC-CM	Reduction in the level of CA-125 and LDH, along with increased apoptosis. Significant decreased of the invasive capacity and aggressiveness of cancer cell lines	[72]
Szyposzynska, A., 2020	MVs from immortalized ADSCs	Reduction in the tumor cell secretion of pro-tumorigenic cytokines, such as IL-6, IL-8, GRO-alpha, and VEGF. Increased levels of tumor-suppressive cytokines such as IL-1ra, IL-2, IL-2Ra, IL-12-p40, IL12-p70, IL-15, and IFN-γ	In vitro on ES-2 and OAW-42 cell culture	MVs were efficiently internalized by both cell types with a decrased metabolic rate and an increased apoptosis. Increased expression of anti-tumor factors in both cell lines compared to control	[73]
**Drug Preloaded ADSCs**					
Melzer, C., 2020	Immortalized ADSCs	ADSCs incubated with taxol and epirubicin internalized the drugs and released drug loaded Exo in the extracellular space with higher cytotoxic activity that the free drugs	In vitro SKOV3 cell lines. In vivo, subcutaneous tumor in SCID mice	Drug-loaded exosomes have a higher cytotoxic effect at lower concentrations than free taxol in vitro. Higher tumor-suppressing capacity, when administered IV in the animal model.	[74]
Borghese, C., 2020	PTX primed ADSCs and PTX-primed ADSC-CM	ADSCs primed with PTX could internalize and release the drug in the extracellular space.	In vitro, SKOV3 2D cultures and 3D spheroid systems	ADSCs primed with PTX and their CM demonstrated a similar tumor-inhibiting capacity. Cytotoxicity was higher than with free PTX and could overcome PTX resistance, both with 2D and 3D culture systems.	[75]
**ADSCs in Enzyme/Prodrug Systems**					
Toro, L., 2016	yCD:UPRT-expressing ADSCs	Adenoviral transduction of yCD:UPRT gene in the ADSCs. ADSC-expressed yCD:UPRT converted 5-FC to 5-FU with high tumor cytotoxic effects	SKOV3 and A2780 cell cultures in vitro. In vivo, intraperitoneal ovarian tumor in nude mice	yCD:UPRT-ADSCs/5-FC combination was efficacious both in 2D and 3D culture systems and both on SKOV3 and A2780 cells. Long-term tumor-free survival in 33% of the animals..	[76]
Malekshah, O.M., 2019	shCE2-expressing ADSCs	Plasmid trasfection of ADSCs with shCE2 gene. Co-administration of IRI and shCE2-expressing ADSCs. Localization of the ADSCs in the tumor site and conversion of IRI to its highly cytotoxic form SN-38, is carried out by the ADSC-secreted shCE2	In vitro, in OVASC-1 cells. In vivo, intraperitoneal ovarian tumor in nude mice	IRI co-administered with shCE2-expressing ADSCs inhibited the proliferation of ALDH+, drug-resistant, ovarian cancer cells at lower concentrations IRI with shCE2-ADSCseradicated ovarian cancer in 80% of the mice, with reduced toxicity,	[77]
Yin, P.T., 2016	TRAIL-expressing ADSCs	Magnetic NP delivered into ADSCs a heat-inducible gene vector that encoded for TRAIL. As a result, mild magnetic hyperthermia-induced TRAIL expression in ADSCs end secretion in the tumor microenvironment	A2780 cell cultures in vitro. In vivo, intraperitoneal ovarian tumor in nude mice	TRAIL-ADSCs, efficiently killed ovarian tumor cells in vitro. Preferential ADSC homing in the tumor after intraperitoneal injection in a mouse model of metastatic ovarian cancer. Efficient TRAIL delivery and significant decrease in tumor volume.	[78]
Kuroki, L.M., 2017	TR3-expressing ADSCs	Adenoviral transduction of TRAIL-based molecule TR3. ADSC expretion of TR3 and secretion in the tumor microenvironment	OVCAR3 cell lines in vitro	Transduced ADSCs efficiently expressed TR3 and secreted it in the extracellular space. TR3-transduced ADSCs and their CM inhibited ovarian cancer cell growth in vitro.	[79]
**ADSCs Combined with Oncolytic Virus**					
Mader, E.K., 2013	Measles virus infected ADSCs	ADSCs localized in the tumor stroma ad released the Measles virus with an oncolytic effect	SKOV3 cell cultures in vitro. In vivo, intraperitoneal ovarian tumor in nude mice	ADSCs efficiently delivered the Measles virus to the tumor stroma, obtaining a cancer-cytotoxic effect and significantly prolonged survival in the treated animals.	[80]

## Data Availability

No new data were created or analyzed in this study. Data sharing is not applicable to this article.
